# Frequency and Management of Accidental Incidents in Orthodontics

**DOI:** 10.3390/children9121801

**Published:** 2022-11-23

**Authors:** Ioannis A. Tsolakis, Isidora Christopoulou, Kalliopi Siotou, Antigoni Alexiou, Vasilis Psarras, Erofili Papadopoulou, Apostolos I. Tsolakis

**Affiliations:** 1 Department of Orthodontics, School of Dentistry, Aristotle University of Thessaloniki, 54124 Thessaloniki, Greece; 2 Department of Orthodontics, National and Kapodistrian University of Athens, 15772 Athens, Greece; 3 Department of Stomatognathic Physiology, Orofacial Pain Clinic, National and Kapodistrian University of Athens, 15772 Athens, Greece; 4 Department of Oral Medicine & Pathology and Hospital Dentistry, School of Dentistry, National and Kapodistrian University of Athens, 10679 Athens, Greece; 5 Department of Orthodontics, Case Western Reserve University, Cleveland, OH 44106, USA

**Keywords:** orthodontics, accidental incidents, clinical practice, accidents

## Abstract

Background: The present study aims to define through questionnaires the frequency and the variety of accidental incidents occurring in orthodontic clinical practice among Greek practicing orthodontists. Methods: A questionnaire survey was conducted among orthodontists from the registry of orthodontists in Greece. The questionnaire was divided into two parts. The first part involved three questions relating to the socio-demographic status and the background of the orthodontist, and the second part concerned exclusively the frequency of accidental incidents that have occurred during clinical practice with three possible answers: never, once, more than once. Results: From the 200 initially distributed questionnaires, 124 were finally completed and sent back (response rate: 62%). The results showed that orthodontists with more years of clinical practice had faced more accidental incidents. Among the ingestion incidents caused by foreign objects, the most frequently occurring was the ingestion of elastic separators, followed by the ingestion of elastic ligatures and ingestion of hooks. The most commonly reported traumatic incidents were the trauma-lesion of the mucosa by the orthodontic wire or part of it, followed by trauma-lesion by hooks and wire ligatures. The reported number of incidents with further complications and with patients referred to an emergency room was very low. Conclusions: The results of the present study determined a high frequency of accidental incidents among Greek orthodontists. The longer clinical experience was accompanied by more accidental incidents. Orthodontists, like other health professionals, must learn and continuously update their knowledge regarding the management protocols of medical emergencies.

## 1. Introduction

Patients’ safety should always be the priority in a dental practice. Unfortunately, published evidence indicates that significant numbers of patients are unintentionally but avoidably harmed in dental offices worldwide [1]. Foreign body ingestion or aspiration episodes are possible complications in all fields of dentistry [1]. Ingestion is a common clinical problem in which an ingested foreign body originating outside the body is ingested into the mouth and through the gastrointestinal tract, while aspiration refers to the introduction of solid matter into the airway at the level of the glottal opening, larynx, trachea, or bronchi [1]. As in orthodontics, the majority of patients are children, and an episode of ingestion or aspiration may be hard to manage. A marked difference in orthodontics, with regard to the other dental specializations, is the frequent use of intraoral appliances, which increases the risk of ingestion episodes. There are multiple appliances that are used in orthodontic treatment, with lots of small pieces inserted directly into the mouth or in parts. Nowadays, those appliances can be customized and fabricated using 3D printing technology [2,3]. In this way, the fitting is perfect, which is highly beneficial and probably diminishes the chances of breakage or detachment of parts during the insertion.

During dental care, as well as during orthodontic appointments, patients are commonly placed in a supine position to enhance access to the oral cavity and improve the comfort of both patient and clinician. In this position, the risk of objects entering the oropharynx is increased [4,5]. The combination of the patient’s position, the liquid oral environment due to saliva, and the handling of small orthodontic components, such as small pieces of wire, orthodontic brackets, and coil springs, poses risks of ingestion of foreign bodies during the orthodontic treatment. It should be highlighted that the aspiration or ingestion of foreign objects during orthodontic treatment can lead to serious medical issues, requiring immediate care and even, in some cases, hospitalization [6]. The incidence of aspiration or swallowing of foreign bodies of dental origin varies considerably among the articles in the published literature. Tamura et al. reported that the range varied considerably from 3.6% to 27.7% for all foreign bodies, with the number being significantly higher in adults than in children [7]. The cases that have been documented concern the swallowing of mandibular spring retainers, expansion appliance keys, fragments of removable maxillary appliances, transpalatal arches (TPA), fractured twin block appliances, and pieces of archwire [8,9,10,11,12,13,14,15]. Fixed or removable orthodontic appliances are worn continuously by patients, and, as a result, accidents frequently occur in or outside the orthodontist’s office. Orthodontic patients may also be at risk of trauma or mucosa lesions, in or outside the orthodontic office, if components become detached or fractured [16].

Most of these incidents were managed conservatively and did not cause serious medical issues. However, a case has been reported in which a 13-year-old boy with Down syndrome accidentally swallowed a removable quad-helix, and surgical intervention was needed [17]. Although there are no reported deaths in the domain of orthodontics as a complication following ingestion or inhalation of foreign bodies, one fatal ingestion of a prosthesis has been documented [18].

According to Clerf, once a foreign object passes the base of the tongue, there is a 12:1 chance that it will settle in the gastrointestinal tract rather than in the airway [19]. The reflexive coughing that occurs when a foreign item is in the patient’s airway, decreasing the likelihood of aspiration, can be used to explain why ingestion happens more frequently than inhalation. Once the foreign body has reached the stomach, there is a greater than 90% chance of the object passing from the gastrointestinal tract without further problems. However, an impacted foreign body in the gastrointestinal tract may also lead to long-term complications such as esophageal erosion or perforation of the gut [20,21,22]. The swallowing of foreign bodies during orthodontic treatment is an adverse effect that, apart from the medical risks that the patient can be exposed to, also creates economic implications and the risk of malpractice litigation.

### Objective

Prevention is obviously the best approach, but the correct management of such an incident, when it occurs, is crucial to the health and safety of the patient and the professional behavior of the clinician. The present study aims to define through questionnaires the frequency and the variety of accidental incidents occurring in orthodontic clinical practice among Greek orthodontists. An algorithm is also proposed to summarize the guidelines that should be followed in order to prevent and manage orthodontic accidental incidents.

## 2. Materials and Methods

### 2.1. Study Design

This cross-sectional study was conducted during 2016–2017 at the Orthodontic Department of the National and Kapodistrian University of Athens, Greece. A comprehensive questionnaire was used to conduct this cross-sectional study. The questionnaires were distributed by e-mail and, in some cases, sent via the post office, along with an explanatory letter to Greek orthodontists, who were asked to complete it and send it back. Participation in the study was voluntary and was not granted by any institution or by the authors of the article. The orthodontists’ names were obtained from the registry of Orthodontists in Greece. Their contact addresses were collected from the Orthodontic Department of the School of Dentistry of Athens and the Greek orthodontic associations. One reminder was mailed to all participants three weeks after sending the questionnaire. Responses were collected anonymously and were concealed. Blinding of the participants’ names was performed. The cover letter explained the rationale for the study, the number of questions, and the contact information for possible explanations and assured the protection of the clinician’s answers and the anonymity of the participants.

### 2.2. Informed Consent and Ethical Clearance

In order to carry out this study, we followed the guidelines of the research ethics committee of the School of Dentistry of the National and Kapodistrian University of Athens. Ethical approval was not required since we investigated the frequency of incidents during orthodontic practice, keeping anonymity without seeking patients’ or orthodontists’ private or sensitive data. Moreover, responses were kept confidential.

### 2.3. Study Details

The questionnaire was divided into two parts. The first part involved three questions relating to the socio-demographic status and the background of the orthodontists. Specifically, the orthodontists were questioned about their years of clinical experience/practice in the field of orthodontics, the University and the country from which they obtained the orthodontic specialization, and the presence or the absence of orthodontic dental assistant(s) or dental staff in their orthodontic office. The second part, with twenty-one questions, was concerned exclusively with the frequency of accidental incidents that had occurred during the orthodontists’ clinical practice. Orthodontists were asked to choose one of three possible answers according to the frequency of incidents: never occurred, occurred once, or occurred more than once. The incidents that were mentioned on the questionnaire were separated into two categories: (a) ingestion incidents and (b) traumatic incidents during the orthodontic treatment. More specifically, the ingestion incidents included ingestion of (a) elastic separator, (b) band, (c) rapid palatal expander, (d) impression material, (e) wire (or part of it), (f) elastic ligatures, (g) wire ligatures, (h) removable appliance for retention, (i) fixed appliance for retention, and (j) hooks. The traumatic incidents during the orthodontic treatment referred to trauma-lesion by (a) headgear, (b) functional appliance for correction of class II, (c) wire (or part of it), (d) elastic ligature, (e) wire ligature, (f) band, (g) hooks, (h) headgear during its adjustment, (i) removable appliance for retention, and (j) orthodontic wire during its adjustment. The last question requested the orthodontists to report any other accidental incident they had that was not mentioned previously in the questionnaire. The questionnaire is presented in the Supplementary Materials.

### 2.4. Statistical Analysis

All data summaries and statistical analyses were performed using the program SPSS Version 23. Univariate chi-square tests were performed at a 5% significance level to test for significant associations between the number of patients experiencing ingestions or inhalation incidents and the socio-demographic factors (year of graduation-clinical practice, country of specialization) among the participating orthodontists.

## 3. Results

**Figure 1:**
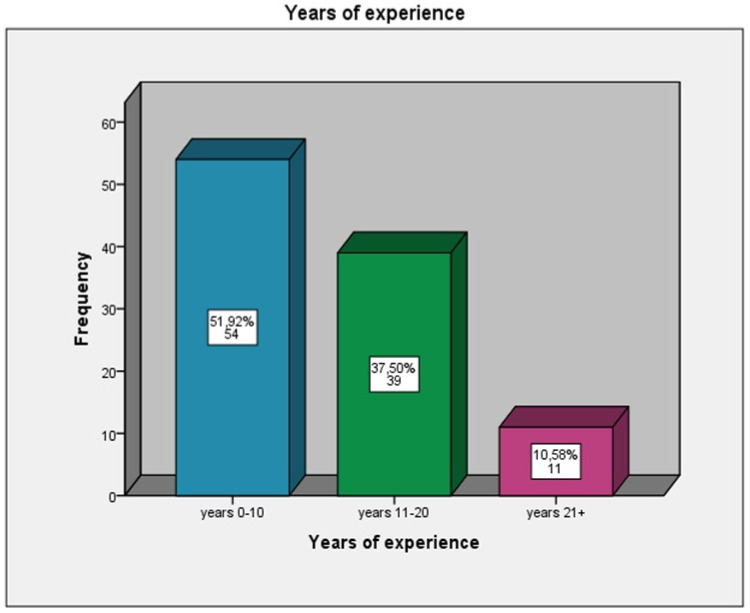
Years of clinical experience in orthodontics.

**Figure 2:**
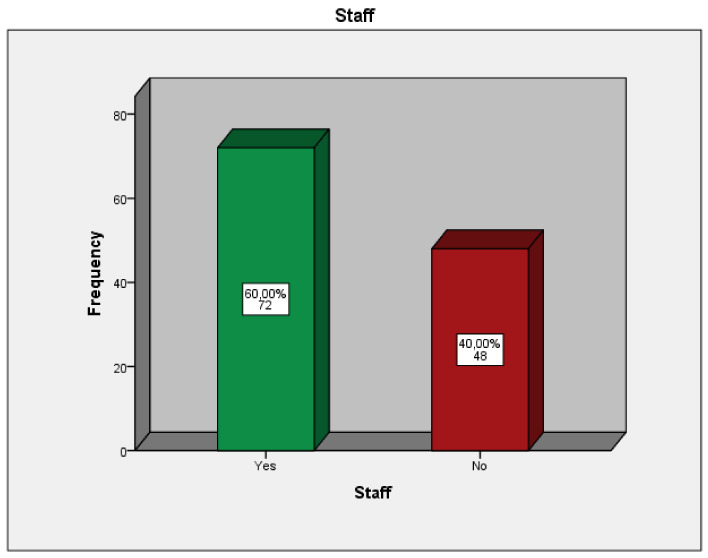
Presence or absence of dental assistant(s)-staff in the orthodontic office.

**Figure 3:**
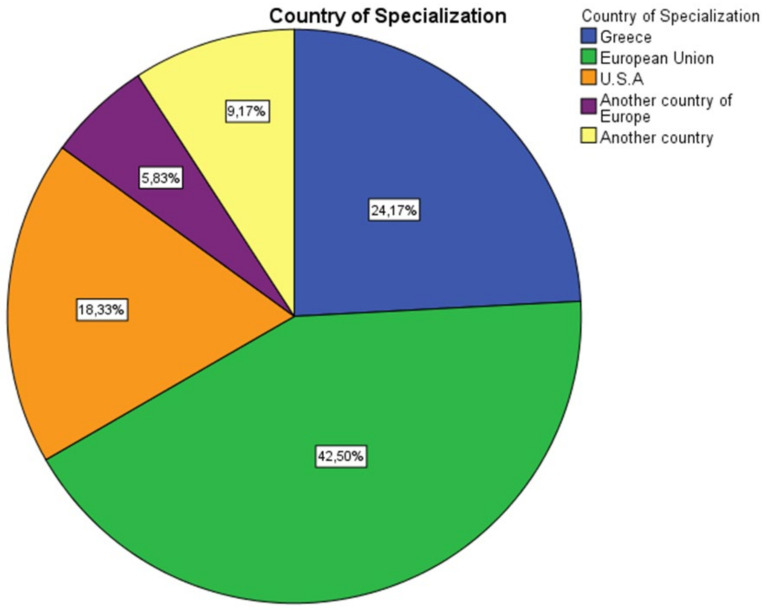
Country of orthodontic specialization.

The total number of the distributed questionnaires was 200; 124 were completed (response rate 62%), sent back, and analyzed. Regarding the socio-demographic characteristics of the respondents, the clinical experience of the orthodontists who participated in the study was in a range of 0–10 years at a percentage of 51.92%,11–20 years at 37.50% of the participants, and 21-plus years of clinical experience at 10.58% of the orthodontists (Figure 1). The majority of the orthodontists have clinical dental staff-assistants in the orthodontic office (60%) (Figure 2) and have obtained a specialization in orthodontics from a country of the European Union (42.50%), Greece (24.17%), or the United States of America (USA) (18.33%) (Figure 3).

**Figure 4:**
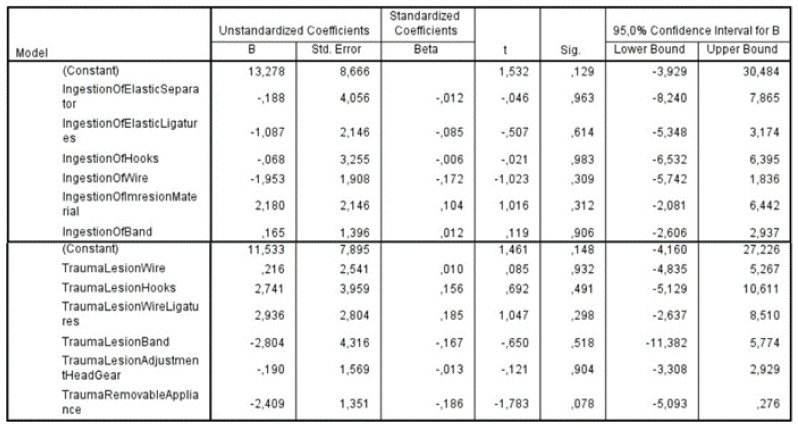
Multiple linear regression for the potential correlation of the factors under investigation with years of practice/experience.

The potential correlation of the factors under investigation with the years of practice/experience was examined with the application of multiple linear regression. The dependent variable was defined as the years of practice. No statistically significant correlations were found between the dependent variable and the independent factors of trauma and ingestion incidents, respectively. These results showed that years of experience in clinical practice was not a critical factor in the number of ingestion and accidental incidents. (Figure 4). Among the ingestion incidents caused by a foreign object, the most frequently occurring were ingestion of an elastic separator (37.19%), followed by ingestion of elastic ligatures (36.89%), and then by ingestion of hooks (28.93%). The above-mentioned incidents occurred, as reported by the orthodontists, more than one time in their dental offices. There was no report of ingestion of the removable appliance, but an individual case of ingestion of a hyrax appliance was mentioned.

**Figure 5:**
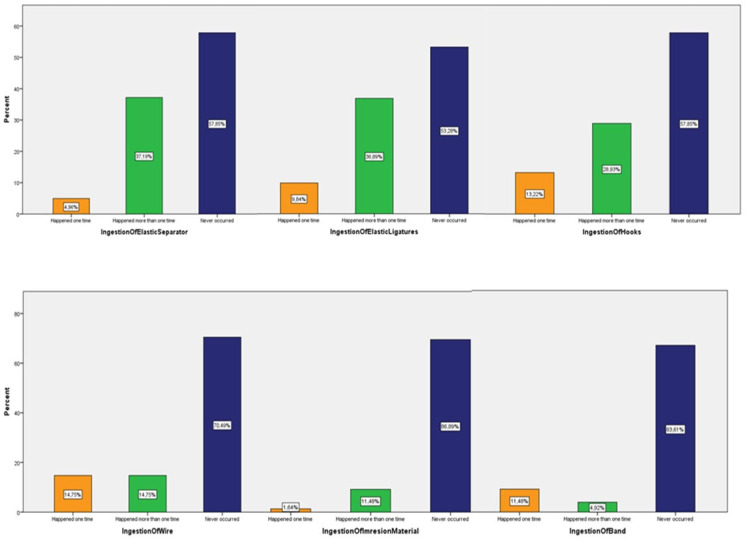
Ingestion incidents.

**Figure 6:**
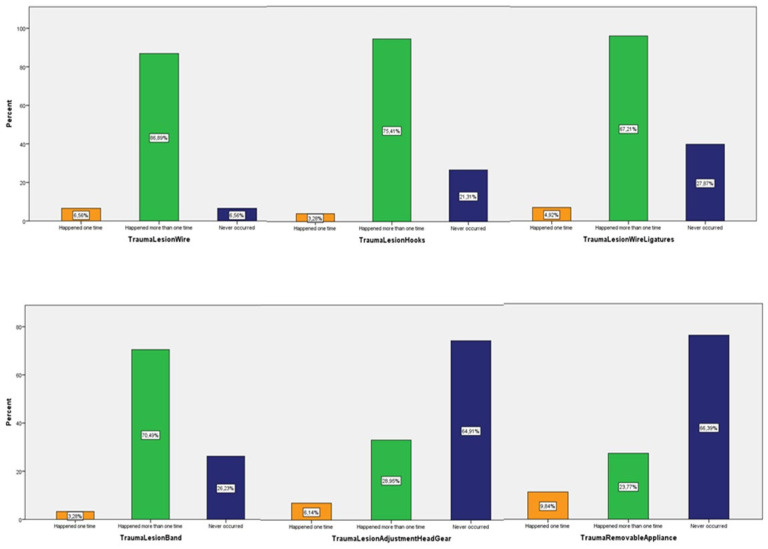
Traumatic incidents.

**Figure 7:**
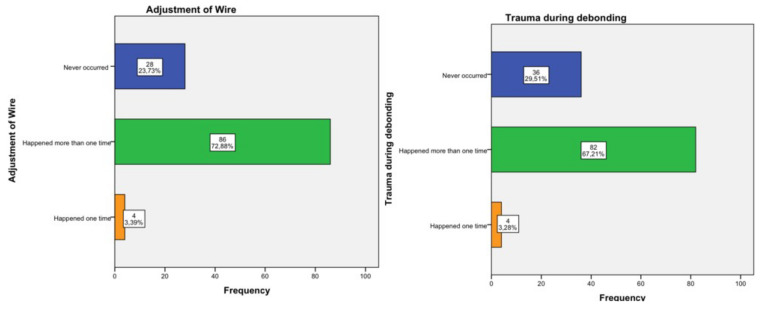
Other incidents.

The most frequently reported traumatic incidents were the trauma-lesion of the mucosa by the orthodontic wire or part thereof (86.89%), followed by the trauma-lesion caused by hooks (70.49%), and then by wire ligatures (67.21%). These incidents occurred more than once, as the records demonstrated. An incident that was also frequently reported at a percentage of 72.88% was trauma during the adjustment of the orthodontic wire at the clinical practice. Furthermore, an individual case of parotid duct obstruction was reported due to the insertion of the band’s hook into the parotid gland, which was treated conservatively, and no further emerging medical complications were mentioned. The results are presented in detail in Figure 5, Figure 6 and Figure 7.

## 4. Discussion

Our study demonstrates that regardless of years of experience, most orthodontists have experienced accidental incidents while practicing their specialty. The fact that orthodontists with more clinical experience reported more cases than orthodontists with fewer years of experience is logical since the former have worked for a longer period and presumably treated more cases with more possibilities for accidental incidents. Varho and colleagues, in a similar study with questionnaires, focused on the frequency of inhalation and ingestion episodes involved in orthodontic treatments in Finland [23]. They found that 20% of dentists and 6.9% of dental hygienists had one experience of inhaling orthodontic objects [23]. The percentages for two or more cases during one’s clinical practice were 18.6% for dentists and 6.9% for dental hygienists [23]. In contrast, we found that the probability of orthodontists experiencing an accidental incident more than once was higher. We think that the difference between the findings of our study and the ones of Varho et al. is rational since, in our study, the questionnaires were answered by orthodontists, while in the study of Varho et al. they were completed by dentists and dental hygienists, who are not specialized in orthodontics and consequently treat fewer orthodontic cases.

Based on the results of our study, the most frequent incident during orthodontic treatment is trauma-lesion of the mucosa by the orthodontic wire or a part thereof. Baricevic et al. agreed with our results, reporting that oral mucosal lesions are frequently seen in the orthodontic population compared to non-orthodontic patients [24]. According to Travess et al., the emergence of ulcers in patients with fixed orthodontic appliances results from the irritation caused by the archwire and bands or from the position of the wire near the lips [25].

The most frequent ingestion incident in our study concerned the elastic separator. The band ingestion frequency was found to be 4.92%, with only 6 out of the 124 participants having such an experience in their professional careers. In a case report concerning the ingestion of an orthodontic band by a treated cleft palate patient, it is mentioned that patients with clefts have a shorter soft palate than non-cleft patients, and the possibility of ingestion or aspiration of a foreign body is relatively higher [26]. Dibiase et al. recommend the use of floss tied through the tube to eliminate the danger of band swallowing during the selection of size for the banding [22]. The existing scientific literature reports that 85% of inhalation and ingestion incidents occurred outside the dental office and without further medical complications. It should be highlighted at this point that, compared to the emergency protocols recommended in the literature [8,22], in this study, the reported number of incidents with further complications and a need for urgent medical care, and the number of patients referred to an emergency room, were very low.

Usually, patients are unaware of the orthodontic appliance’s breaking and the possibility of having swallowed part of it, and the orthodontist is the first to notice it [27]. During orthodontic treatment, which usually lasts from two to three years, patients are required to have a variety of removable and fixed appliances with many little components. Reis et al. report that dental practitioners and orthodontic specialists may not even wonder about or be concerned about the possibility of the patient swallowing something due to the high frequency of detached orthodontic brackets, wires, or appliances [28]. At this point, it is important to highlight the increased failure rate in the adhesion of orthodontic brackets as a predisposing factor (up to 7.4% to 10.6% in a 6-month period and 15.6% to 17.6% in an 18-month period) [29].

In an attempt to avoid any traumatic lesion, researchers recommend that removable appliances should be smooth and rounded and the acrylic colored any color other than pink to facilitate visual recognition during bronchoscopy or endoscopy to locate the acrylic fragments if they are inhaled or swallowed [22,30].

### 4.1. Limitations and Recommendations

This research is an original work, as never before has the frequency of accidental incidents among Greek orthodontists been studied. The limitations of this cross-sectional study include the lack of complete information on whether the injuries occurred during actions performed by the orthodontist or the dental staff. This is a domain that should be further investigated as it is more probable for an incident to occur during handling by the dental assistant than in the hands of the experienced orthodontist. Another limitation arises from the fact that approximately half of the registered Greek orthodontists took part in the study. The participants volunteered for the study, which could result in voluntary bias. Moreover, as far as the limitations regarding patients are concerned, the participating orthodontists did not provide us with adequate information concerning the age, medical history, and possible syndromes affecting patients’ communication levels in cases of inhalation or ingestion. It is rational to conclude that the possibly lower level of communication-cooperation between patient and clinician increases the possibility of accidental incidents. Further, children’s emerging maturity age largely affects their level of cooperation. This information would be a valuable asset in future research in this direction.

### 4.2. Management of Incidents in Orthodontic Practice

The emergency protocols for foreign body ingestion or aspiration are the ones mostly followed by the medical specialties of gastroenterology, respiratory medicine, and emergency medicine, and much less by dental specialties. [31,32]. The orthodontic specialty, as well as all the dental community, is in high need of established, up-to-date emergency protocols. From our personal experience and after analyzing the questionnaires in detail, we have concluded that the risk of perforation or obstruction depends on the size and shape of the objects and is higher for a sharp object. Unfortunately, the majority of orthodontic components are small, sharp, and often difficult to handle. The risk of obstruction also increases in the presence of anatomical anomalies, such as constrictions and roughness. Here are presented some general remarks that we have established over the years and that clinicians may find useful in attempting to eliminate the possibilities of orthodontic accidental incidents. These remarks are in accordance with the existing management guidelines published in the literature [31,32].

- Do not use inefficient, inappropriate, or damaged instruments while cutting the end of the wire.
- Use compatible cement to attach brackets and bands.
- Use temporary loops made of dental floss attached to orthodontic appliances during adjustment and manipulation in the orthodontic office.
- Give further attention and time to patients with syndromes or other problems affecting their communication level.
- In case of ingestion, laxatives are not useful and may even increase the risk of perforation.

Based on our guidelines, the most important is for the orthodontist and the staff members to be informed about the danger the patient can be exposed to in cases of inhalation or ingestion of orthodontic appliance objects and to always be prepared to intervene. They must have the proper emergency equipment, written emergency procedures, and the telephone number of a physician and of the local medical emergency unit. Orthodontists and team members should also periodically participate in medical emergency management courses. Patients and parents should also be informed before the beginning of the orthodontic treatment of all the possible adverse effects patients may experience during the course of their treatment. Just before the patient leaves the office, the orthodontist should evaluate the orthodontic appliances and mechanisms to identify any breakage of wires or loss of attachments. If the patient feels that an object fell on the tongue, he or she should try to suppress the swallowing reflex and turn the head to the side. Any foreign object suspected of being ingested or inhaled should be localized radiographically as soon as possible, and the decision to manage it conservatively or surgically must be taken with caution.

## 5. Conclusions

Our study determined a high frequency of accidental incidents among Greek orthodontists. The majority of them concerned traumatic incidents of the mucosa, while there were fewer ingestion incidents reported. Longer clinical experience was accompanied by more accidental incidents. In such incidents, the precautions taken and the preparedness on the part of the orthodontist in managing these episodes are what actually mattered. The orthodontist and the assistant staff must be trained and capable of providing basic life support measures. Orthodontists, like other health professionals, must learn and continuously update their knowledge regarding the management of medical emergencies.

## Data Availability

The datasets used and/or analyzed during the current study are available from the corresponding author upon reasonable request.

## Supplementary Materials

The following supporting information can be downloaded at: https://www.mdpi.com/article/10.3390/children9121801/s1.
