# Supplementary material for: Frequency and Management of Accidental Incidents in Orthodontics
Source: Children (Basel). 2022 Nov 23;9(12):1801. doi: 10.3390/children9121801 (PMC9777126; doi:10.3390/children9121801)
Supplement: Supplementary file 1 [file children-09-01801-s001.zip › children-1994089-supplementary.pdf]

HELLENIC REPUBLIC
National and Kapodistrian
University of Athens
EST. 1837

SCHOOL OF DENTISTRY
2, THIVON STR. GOUDI
115 27 ATHENS -GREECE
TEL 210-7461228 – FAX 210-7461213

How many years have you been practicing orthodontics? ……….

In which country have you been trained?

- ☐ Greece
- ☐ European Union
- ☐ USA
- ☐ Another country of Europe
- ☐ Another country

Do you have dental assistant(s)/staff in your doctor's office?

- ☐ Yes
- ☐ No

If you answered yes, how many people do you employ? ……….

Which of the following orthodontic procedures do you perform alone, which with your dental assistant and which are performed exclusively by your assistant?
In cases where the procedure is performed by the orthodontist or the assistant, please check both.

Procedure	Orthodontist	Orthodontist with dental assistant	Dental assistant
1. Placement of elastic separator			
2. Placement of band			
3. Taking photo			
4. Taking impression			
5. Placement of wire			
6. Placement of elastic ligature			
7. Placement of wire ligature			
8. Placement of headgear			
9. Placement of removable appliance			
10. Placement of fixed appliance			
11. Placement of bracket			
12. Placement of functional appliance for Class II correction			
13. Removal of elastic separator			
14. Removal of wire			
15. Removal of elastic ligature			
16. Removal of wire ligature			

17. Removal of band			
18. Removal of bracket			
19. Headgear adjustment			
20. Fixed appliance adjustment			
21. Adjustment of functional appliance for Class II correction			
22. Wire adjustment (bending)			

Which and how often have the following incidents occured to you?

Incident	Once	More than once	Never
1. Ingestion of elastic separator			
2. Ingestion of band			
3. Ingestion of rapid palatal expander			
4. Ingestion of impresion material			
5. Ingestion of wire			
6. Ingestion of elastic ligature			
7. Ingestion of wire ligature			
8. Trauma lesion by headgear			
9. Ingestion of removable appliance			
10. Ingestion of fixed appliance			
11. Ingestion of hooks/brackets			
12. Trauma lesion by functional appliance for Class II correction			
13. Trauma lesion by wire			
14. Trauma lesion during the removal of elastic ligature			
15. Trauma lesion by wire ligature			
16. Trauma lesion by band			
17. Trauma lesion by hook/bracket			
18. Trauma lesion during headgear adjustment			
19. Trauma lesion by removable appliance			

20. Trauma lesion during wire adjustment			
21. Record any other incident has happened to you			
22. Record any other incident has happened to you			
23. Record any other incident has happened to you			
